# Structural insights into protection against a SARS-CoV-2 spike variant by T cell receptor diversity

**DOI:** 10.1016/j.jbc.2023.103035

**Published:** 2023-02-17

**Authors:** Daichao Wu, Grigory A. Efimov, Apollinariya V. Bogolyubova, Brian G. Pierce, Roy A. Mariuzza

**Affiliations:** 1 Laboratory of Structural Immunology, Department of Hepatopancreatobiliary Surgery, The First Affiliated Hospital, Hengyang Medical School, University of South China, Hengyang, Hunan, China; 2 W.M. Keck Laboratory for Structural Biology, University of Maryland Institute for Bioscience and Biotechnology Research, Rockville, Maryland, USA; 3 Department of Cell Biology and Molecular Genetics, University of Maryland, College Park, Maryland, USA; 4 National Research Center for Hematology, Moscow, Russia

**Keywords:** T cell receptor, SARS-CoV-2, spike epitope, viral escape, X-ray crystallography, CDR, complementarity-determining region, CP, convalescent patient, pMHC, peptide–MHC, SARS-CoV-2, severe acute respiratory syndrome coronavirus 2, TCR, T cell receptor, VOC, variant of concern, Vα, variable α, Vβ, variable β

## Abstract

T cells play a crucial role in combatting SARS-CoV-2 and forming long-term memory responses to this coronavirus. The emergence of SARS-CoV-2 variants that can evade T cell immunity has raised concerns about vaccine efficacy and the risk of reinfection. Some SARS-CoV-2 T cell epitopes elicit clonally restricted CD8^+^ T cell responses characterized by T cell receptors (TCRs) that lack structural diversity. Mutations in such epitopes can lead to loss of recognition by most T cells specific for that epitope, facilitating viral escape. Here, we studied an HLA-A2–restricted spike protein epitope (RLQ) that elicits CD8^+^ T cell responses in COVID-19 convalescent patients characterized by highly diverse TCRs. We previously reported the structure of an RLQ-specific TCR (RLQ3) with greatly reduced recognition of the most common natural variant of the RLQ epitope (T1006I). Opposite to RLQ3, TCR RLQ7 recognizes T1006I with even higher functional avidity than the WT epitope. To explain the ability of RLQ7, but not RLQ3, to tolerate the T1006I mutation, we determined structures of RLQ7 bound to RLQ–HLA-A2 and T1006I–HLA-A2. These complexes show that there are multiple structural solutions to recognizing RLQ and thereby generating a clonally diverse T cell response to this epitope that assures protection against viral escape and T cell clonal loss.

The global coronavirus disease 2019 (COVID-19) pandemic is caused by severe acute respiratory syndrome coronavirus 2 (SARS-CoV-2) (1, 2, 3). Elucidating the mechanisms underlying the adaptive immune response to SAR-CoV-2 is crucial for predicting vaccine efficacy and assessing the risk of reinfection. The emerging consensus is that neutralizing antibodies, CD4^+^ helper T cells, and CD8^+^ killer T cells all contribute to the control of SARS-CoV-2 and the protection offered by current vaccines, although precise immune correlates of protection remain to be defined (4, 5, 6). Neutralizing antibodies against SARS-CoV-2 are clearly protective but may be short-lived and are not elicited in all infected individuals (7). Mounting evidence indicates that T cells play a vital role in the clearance of SARS-CoV-2 and in formation of long-term memory responses to this virus (5, 6, 8, 9). For example, the observation that individuals with agammaglobulinemia or pharmaceutical depletion of antibody-producing B cells generally experience an uncomplicated COVID-19 disease course suggests that T cells are able to mount an effective response against SARS-CoV-2 even when antibody responses are absent or inadequate (10, 11, 12, 13). Also consistent with an important contribution of T cells to recovery from COVID-19 are numerous reports of healthy individuals successfully controlling SARS-CoV-2 infection in the absence of detectable neutralizing antibodies but with prominent SARS-CoV-2–specific T cell memory (5, 14, 15, 16, 17, 18).

The emergence of SARS-CoV-2 variants has raised concerns of escape from vaccine-induced immunity. Variants of concern (VOCs) are associated with a high degree of transmissibility and disease severity. A number of studies have revealed a reduction in antibody-mediated neutralization of Omicron VOCs in vaccinated individuals (19, 20, 21). In addition, there is emerging evidence that SARS-CoV-2 variants can evade T cell immunity (22, 23, 24, 25). In one study, changes in an immunodominant spike-encoded CD8^+^ T cell epitope in Omicron variants BA.1, BA.2, and BA.3 ablated T cell recognition of this HLA-A∗29:02–restricted epitope in COVID-19 convalescent patients (CPs) and vaccinated individuals (23). In another study, a mutation (P272L) in a spike epitope corresponding to residues 269 to 277 (YLQPRTFLL; designated YLQ) that has arisen in >100 different SARS-CoV-2 lineages to date, including VOCs, was not recognized by >175 different individual YLQ-specific T cell receptors (TCRs) isolated from HLA-A∗02:01^+^ COVID-19 CPs and vaccinees (24). Crystal structures of four of these TCRs bound YLQ–HLA-A2 revealed the molecular basis for loss of recognition of the P272L mutant epitope (24, 26, 27, 28).

A striking feature of the T cell response to the YLQ spike epitope is a lack of structural diversity: the large majority (>85%) of HLA-A∗02:01–restricted, YLQ-specific TCRs used the almost identical TRAV12-1 or TRAV12-2 gene segments with prominent CDR3 motifs that were shared across individuals (16, 17, 24). All four TCRs in the TCR–YLQ–HLA-A2 structures employ TRAV12-1 or TRAV12-2 and adopt nearly the same binding strategy in which TCR contacts with peptide are dominated by CDR3α with an important contribution from complementarity-determining region 1α (CDR1α) (24, 26, 27, 28). Substitution of proline 272 with leucine in the P272L variant would create steric clashes with both these CDR loops, thereby abolishing recognition. The restricted structural diversity of YLQ-specific TCRs may facilitate viral escape from T cells targeting this epitope and explain the emergence and transmission of the P272L mutation in multiple SARS-CoV-2 lineages (24).

In sharp contrast to YLQ-specific TCRs, TCRs from COVID-19 CPs specific for a different HLA-A∗02:01–restricted spike epitope corresponding to residues 1000 to 1008 (RLQSLQTYV; designated RLQ) were found to utilize a wide variety of unrelated α/β chain pairs, including TRAV16/TRBV11-2, TRAV12-2/TRVB6-5, TRAV38-2DV8/TRVB12-3, and TRDV1/TRBV20-1 (16, 26). It is believed that such diversity in antiviral T cell responses assures protection against viral escape and the provision of T cell functional heterogeneity (29).

We previously determined the crystal structure of an RLQ-specific TCR (RLQ3) expressing the TRAV16/TRBV11-2 gene combination bound to RLQ–HLA-A2 (26). Here, we report structures of an RLQ-specific TCR (RLQ7) expressing a different α/β chain pair (TRAV38-2DV8/TRVB12-3) from RLQ3 bound to RLQ–HLA-A2 and T1006I–HLA-A2. T1006I is the most common natural variant of the RLQ epitope in the GISAID database (30) and contains a threonine-to-isoleucine mutation at position 1006 (RLQSLQ**I**YV). As measured by surface plasmon resonance, RLQ7 binds T1006I–HLA-A2 with a dissociation constant (*K*_D_) of 62.8 μM, which is nearly identical to its *K*_D_ for WT RLQ (49.0 μM; 1.3-fold affinity reduction) (26). The effect of the T1006I mutation on RLQ3 binding is significantly greater: *K*_D_ = 170 μM for T1006I versus 32.9 μM for WT RLQ (5.2-fold affinity reduction). In agreement with surface plasmon resonance, T cell activation assays (EC_50_) revealed that RLQ7 is much less affected by the T1006I mutation than RLQ3 (26). Whereas RLQ3 showed a 9-fold EC_50_ increase (3.3 μM to 29 μM), RLQ7 showed a 3-fold EC_50_ reduction (0.28 μM to 0.09 μM), indicating that RLQ7 actually has higher functional avidity for the T1006I variant than the WT epitope. Structural analysis of the RLQ7–RLQ–HLA-A2 and RLQ3–RLQ–HLA-A2 complexes demonstrated that there are multiple molecular solutions to recognizing RLQ and thereby generating a clonally diverse T cell response that cannot be easily overcome by SARS-CoV-2 through epitope mutations.

## Results

### Overview of the RLQ7–RLQ–HLA-A2 complex

**Figure 1:**
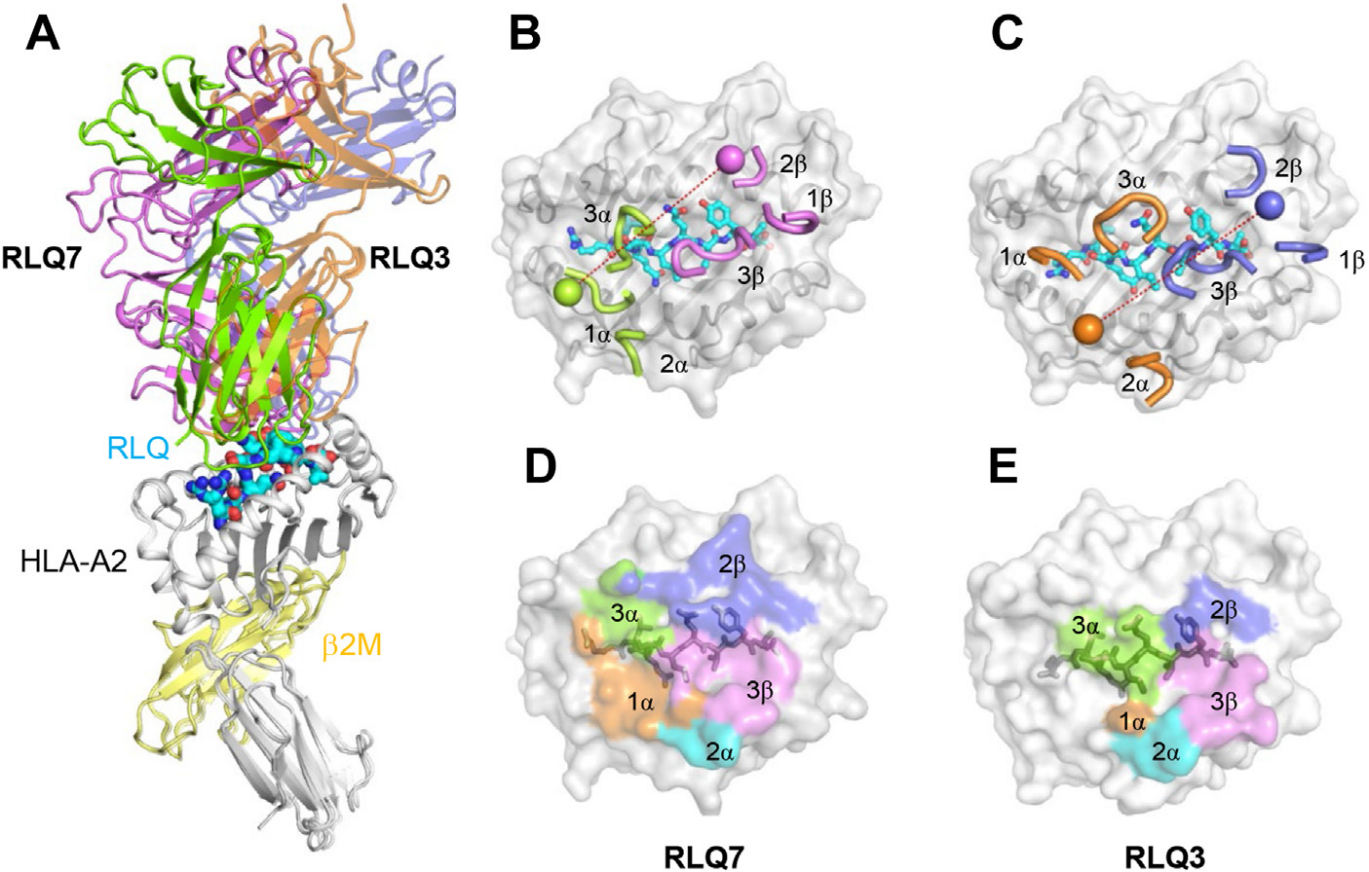
**Structure of TCRs RLQ7 and RLQ3 in complex with RLQ–HLA-A2.***A*, side view of the RLQ7–RLQ–HLA-A2 and RLQ3–RLQ–HLA-A2 complexes (ribbon diagram). The complexes are superposed through their common RLQ–HLA-A2 component. RLQ7 α chain, *green*; RLQ7 β chain, *magenta*; RLQ3 α chain, *orange*; RLQ3 β chain, *blue*; HLA-A2 heavy chain, *gray*; β_2_-microglobulin (β_2_m), *yellow*. The RLQ peptide is *cyan*. *B*, positions of CDR loops of TCR RLQ7 on RLQ–HLA-A2 (top view). CDRs are shown as numbered *green* (CDR1α, CDR2α, and CDR3α) or *magenta* (CDR1β, CDR2β, and CDR3β). HLA-A2 is depicted as a *gray* surface. The *green* and *magenta spheres* mark the positions of the conserved intrachain disulfide of the Vα and Vβ domains, respectively. The *red dashed line* indicates the crossing angle of TCR to pMHC. *C*, positions of CDR loops of TCR RLQ3 on RLQ–HLA-A2 (top view). CDRs are shown as numbered *orange* (CDR1α, CDR2α, and CDR3α) or *blue* (CDR1β, CDR2β, and CDR3β). *D*, footprint of TCR RLQ7 on RLQ–HLA-A2. The top of the MHC molecule is depicted as a *gray* surface. The areas contacted by individual CDR loops are color-coded: CDR1α, *orange*; CDR2α, *cyan*; CDR3α, *green*; CDR2β, *blue*; CDR3β, *magenta*. *E*, footprint of TCR RLQ3 on RLQ–HLA-A2. CDR1α, complementarity-determining region 1α; TCR, T cell receptor; pMHC, peptide–MHC.

TCR RLQ7, like TCR RLQ3, was isolated by screening CD8^+^ T cells from COVID-19 CPs with RLQ–HLA-A2 tetramers (16). However, whereas RLQ7 utilizes gene segments TRAV38-2DV8 and TRAJ29 for the α chain and TRBV12-3 and TRBJ2-3 for the β chain, RLQ3 utilizes TRAV16 and TRAJ39 for the α chain and TRBV11-2 and TRBJ2-3 for the β chain. To understand how two TCRs using completely different α/β chain pairs recognize the same RLQ–HLA-A2 ligand, we determined the structure of the RLQ7–RLQ–HLA-A2 complex to 2.78 Å resolution (Table S1) (Fig. 1*A*). The interface between TCR and peptide–MHC (pMHC) was in unambiguous electron density (Fig. S1*A*).

TCR RLQ7 docks over RLQ–HLA-A2 in a canonical diagonal orientation, with variable α (Vα) over the α2 helix of HLA-A2 and variable β (Vβ) over the α1 helix. The crossing angle of TCR to pMHC (31) is 39°, which is similar to the crossing angle of RLQ3 (36°) (Fig. 1, *B* and *C*). The incident angle (32), which corresponds to the degree of tilt of TCR over pMHC, is 16°, compared to 18° for RLQ3. As depicted by the footprints of RLQ7 and RLQ3 on the pMHC surface (Fig. 1, *D* and *E*), RLQ7 engages RLQ–HLA-A2 mainly through CDR1α (29% of total contacts), CDR2β (31%), and CDR3β (26%), whereas RLQ3 primarily employs CDR3α (37% of total contacts) and CDR3β (31%) to bind pMHC. The buried surface area of the RLQ7 interaction with RLQ–HLA-A2 is 2217 Å^2^, markedly higher than the RLQ3 interface with RLQ–HLA-A2 (1659 Å^2^). Additionally, the RLQ7–RLQ–HLA-A2 interface size is high in comparison with known TCR–pMHC structures, in the top 10% (ranked 15 out of 218) when compared with experimentally determined MHC class I complex structures in the TCR3d database (33).

### Interaction of TCR RLQ7 with HLA-A2

**Figure 2:**
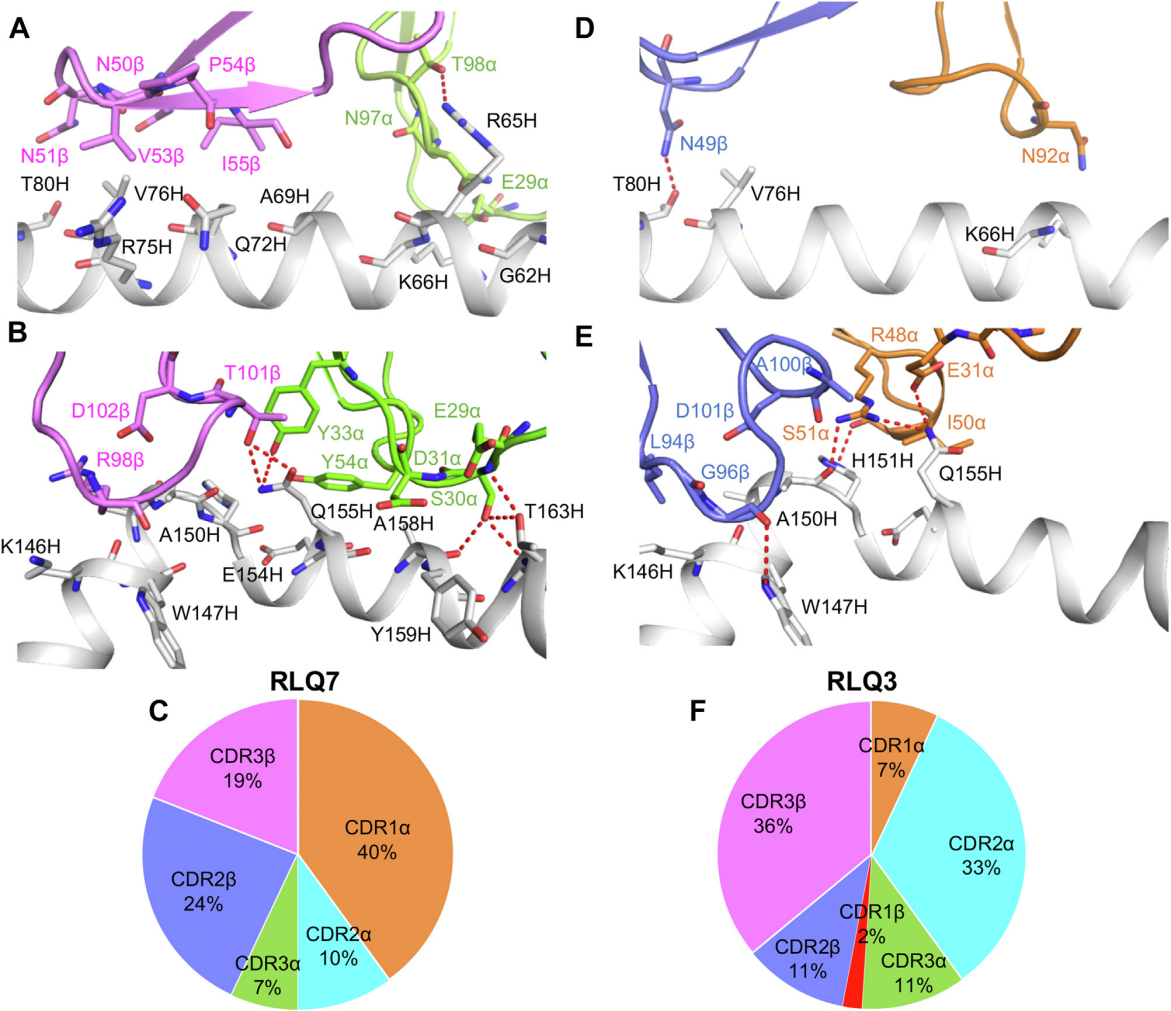
**Interactions of TCRs RLQ7 and RLQ3 with HLA-A2.***A*, interactions between RLQ7 and the HLA-A2 α1 helix. The side chains of contacting residues are drawn in *stick* representation with carbon atoms in *green* (TCR α chain), *magenta* (TCR β chain), or *gray* (HLA-A2), nitrogen atoms in *blue*, and oxygen atoms in *red*. Hydrogen bonds are indicated by *red dashed lines*. *B*, interactions between RLQ7 and the HLA-A2 α2 helix. *C*, pie chart showing percentage distribution of TCR RLQ7 contacts to HLA-A2 according to CDR. *D*, interactions between RLQ3 and the HLA-A2 α1 helix. *E*, interactions between RLQ3 and the HLA-A2 α2 helix. *F*, pie chart showing percentage distribution of TCR RLQ3 contacts to HLA-A2 according to CDR. TCR, T cell receptor.

TCR RLQ7 engages HLA-A2 through interactions distinct from those of RLQ3 (Fig. 2), but with some broad similarities. Of the total number of contacts (68) that RLQ7 makes with HLA-A2, excluding the RLQ peptide, CDR1α, CDR2α, and CDR3α contribute 40%, 10%, and 7%, respectively, compared with 0%, 24%, and 19% for CDR1β, CDR2β, and CDR3β, respectively (Table 1). Hence, the germline-encoded CDR1 and CDR2 loops of RLQ7 dominate interactions with MHC (50 of 68 contacts; 74%), with CDR1α and CDR2β making the greatest contributions (Fig. 2*C*). By contrast, RLQ3 relies on the somatically generated CDR3α and CDR3β loops for MHC recognition to approximately the same extent as the CDR1 and CDR2 loops (26 *versus* 29 contacts) (Table 1) (Fig. 2*F*).

**Table 1:** TCR CDR atomic contacts with peptide and MHC


	α chain				β chain				
	CDR1	CDR2	HV4	CDR3	CDR1	CDR2	HV4	CDR3	Total^a^
# Of contacts									
RLQ3									
peptide	4	0	0	42	0	12	0	21	79
MHC	4	18	0	6	1	6	0	20	55
RLQ7									
peptide	12	0	0	8	0	26	0	22	68
MHC	27	7	0	5	0	16	0	13	68
% of contacts									
RLQ3									
peptide	5	0	0	53	0	15	0	27	
MHC	7	33	0	11	2	11	0	36	
RLQ7									
peptide	18	0	0	12	0	38	0	32	
MHC	40	10	0	7	0	24	0	19	

Contacts were calculated between nonhydrogen atoms with a 4.0 Å distance cutoff.

a Total contacts reflect the total number of TCR–MHC or TCR–peptide contacts.

Similar to RLQ3, RLQ7 makes only sparse contacts with the HLA-A2 α1 helix, primarily *via* CDR2β (Fig. 2, *A* and *D*). However, RLQ7, like RLQ3, interacts extensively with the HLA-A2 α2 helix *via* CDR1α, CDR2α, and CDR3β (Fig. 2, *B* and *E*), with Tyr33α and Thr101β forming three side-chain–side-chain hydrogen bonds with Gln155H that link the TCR to the central section of helix α2: Y33α Oη–Nε2 Q155 HLA-A2, T101β Oγ1–Nε2 Q155 HLA-A2, and T101β Oγ1–Oε1 Q155 HLA-A2 (Table S2) (Fig. 2*B*). In ,addition, Ser30α establishes four hydrogen bonds with Ala158H and Thr163H at the C-terminus of helix α2 that provide further stabilization: S30α Oγ–O A158 HLA-A2, S30α N–Oγ1 T163 HLA-A2, S30α Oγ–Oγ1 T163 HLA-A2, and S30α Oγ–Ν T163 HLA-A2. By contrast, RLQ3 interacts only with the central section of helix α2 (Fig. 2*E*).

### RLQ epitope recognition by TCR RLQ7

**Figure 3:**
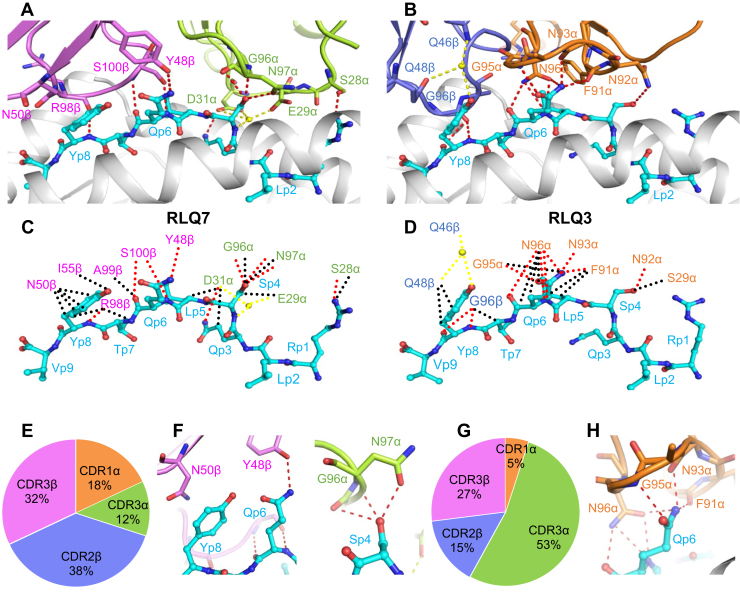
**Interactions of TCRs RLQ7 and RLQ3 with RLQ peptide.***A*, interactions between TCR RLQ7 and the RLQ peptide. The side chains of contacting residues are drawn in *stick* representation with carbon atoms in *green* (TCR α chain), *magenta* (TCR β chain), or *cyan* (RLQ peptide), nitrogen atoms in *blue*, oxygen atoms in *red*, and water molecules as *yellow spheres*. Peptide residues are identified by one-letter amino acid designation followed by position (p) number. Hydrogen bonds are indicated by *red dashed lines*. Water-mediated hydrogen bonds are drawn as *yellow dashed lines*. *B*, interactions between TCR RLQ3 and the RLQ peptide. Carbon atoms are *orange* (TCR α chain), *blue* (TCR β chain), or *cyan* (RLQ peptide). *C*, schematic representation of interactions between TCR RLQ7 and the RLQ peptide. Hydrogen bonds are *red dotted lines* and van der Waals contacts are *black dotted lines*. For clarity, not all van der Waals contacts are shown. *D*, schematic representation of interactions between TCR RLQ3 and the RLQ peptide. *E*, pie chart showing percentage distribution of RLQ7 contacts to the RLQ peptide according to CDR. *F*, close-up of interactions between RLQ7 and P4 Ser, P6 Gln, and P8 Tyr. *G*, pie chart showing percentage distribution of RLQ3 contacts to the RLQ peptide according to CDR. *H*, close-up of interactions between RLQ3 and P6 Gln. TCR, T cell receptor.

Like RLQ3, RLQ7 engages the N-terminal half of the RLQ peptide mainly through the CDR1α and CDR3α loops, whereas the CDR2β and CDR3β loops contact the C-terminal half (Table S3) (Fig. 3, *A*–*D*). However, the specific interactions with RLQ made by the two TCRs are completely different (Table S3). Most contacts between RLQ7 and RLQ are mediated by Vβ (48 of 68; 70%), with CDR2β and CDR3β accounting for 26 and 22 contacts, respectively (Fig. 3*E*). By contrast, CDR3α alone contributes more than half the contacts between RLQ3 and RLQ (42 of 79; 53%) (Fig. 3*G*). TCR RLQ7 engages all seven solvent-exposed residues along the entire length of RLQ (P1 Arg, P3 Gln, P4 Ser, P5 Leu, P6 Gln, P7 Thr, and P8 Tyr), whereas TCR RLQ3 makes no contacts with the N-terminal portion of the peptide (Fig. 3, *C* and *D*) (Table S3 and S4). In contrast to RLQ3, whose principal focus is on P6 Gln, interactions between RLQ7 and RLQ (68 van der Waals contacts and nine hydrogen bonds) are more evenly distributed among accessible peptide residues, thereby enabling maximum readout of the RLQ sequence (Table S4) (Fig. S2).

Computational alanine scanning mutagenesis with the RLQ7–RLQ–HLA-A2 structure supports the change in peptide residues engaged by RLQ7 *versus* RLQ3 (Table S5). Notably, P3 Gln is a predicted hotspot (binding ΔΔ*G* ≥ 1.0 Rosetta Energy Units, which are comparable to kcal/mol, for alanine substitution) for RLQ7 binding, and there is additional predicted energetic involvement, albeit below hotspot level, by residues P4 Ser and P1 Arg. In contrast, there are no predicted hotspots in the N-terminal portion of the peptide for RLQ3 binding. These distinct energetic and contact footprints on the RLQ peptide, with RLQ7 displaying broader engagement of the peptide residues *versus* more C-terminally focused recognition by RLQ3, likely enables RLQ7 to maintain binding to the T1006I variant of RLQ while RLQ3 shows loss of binding. In addition, the greater extent (in proportion to peptide and overall amount) of contacts with the HLA-A2 MHC by RLQ7 (68 MHC contacts and 68 peptide contacts for RLQ7 *versus* 55 MHC contacts and 79 peptide contacts for RLQ3) (Table S4) also likely contributes to its more permissive binding of the T1006I RLQ variant.

### Structural effects of the T1006I mutation on RLQ7 binding

**Figure 4:**
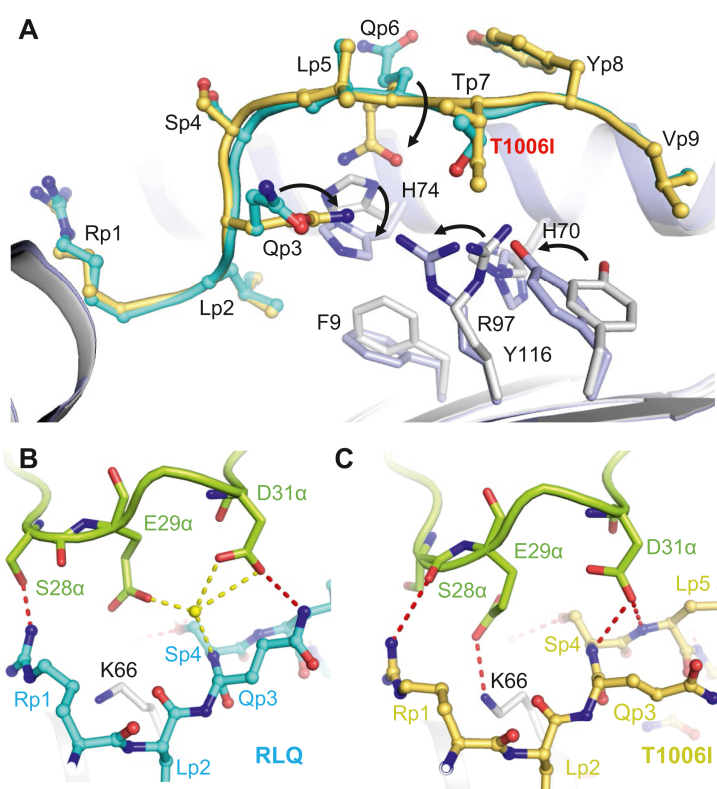
**Comparison of the RLQ7–RLQ–HLA-A2 and RLQ7–T1006I–HLA-A2 complexes.***A*, structural rearrangements in peptide–MHC resulting from the T1006I mutation (RLQ peptide, *cyan*; T1006I peptide, *yellow*; HLA-A2 bound to RLQ, *gray*; HLA-A2 bound to T1006I, *light blue*). *Black arrows* indicate movements in the position of affected residues. *B*, interactions between CDR1α of RLQ7 (*green*) and the RLQ peptide (*cyan*). *Yellow sphere* is an interfacial water molecule. Hydrogen bonds are *red dashed lines*. Water-mediated hydrogen bonds are *yellow dashed lines*. *C*, interactions between CDR1α of RLQ7 (*green*) and the T1006I peptide (*yellow*). CDR1α, complementarity-determining region 1α.

To explain the ability of TCR RLQ7 to tolerate the T1006I mutation, we determined the structure of the RLQ7–T1006I–HLA-A2 complex to 2.60 Å resolution (Table S1) (Fig. S1*B*) and compared it to that of the WT RLQ7–RLQ–HLA-A2 complex. The T1006I substitution, which occurs at secondary anchor position P7, is associated with structural rearrangements in the MHC peptide-binding groove and in peptide residues P3 Gln and P6 Gln (Fig. 4*A*), both of which contact TCR RLQ7 (Table S3). In particular, the side chain of P6 Gln undergoes a 150° flip relative to its position in the RLQ7–RLQ–HLA-A2 structure that redirects the side chain away from RLQ7 and towards HLA-A2. As a consequence, a side-chain–side-chain hydrogen bond linking P6 Gln to TCR in the RLQ7–RLQ–HLA-A2 structure (Tyr48β Oη–Nε2 P6 Gln) is lost; however, two other hydrogen bonds involving main-chain atoms of P6 Gln (Ser100β Oγ–N P6 Gln and Ser100β N–O P6 Gln) are retained (Table S3). In the RLQ7–RLQ–HLA-A2 complex, CDR1α engages the N-terminal region of RLQ *via* two direct (Ser28α Oγ–Nη1 P1 Arg and Asp31α Oδ2–Nε2 P3 Gln) and three water-mediated (Glu29α Oε2–H_2_O–N P4 Ser, Asp31α Oδ1–H_2_O–N P4 Ser, and Asp31α Oδ2–H_2_O–N P4 Ser) hydrogen bonds (Fig. 4*B*). However, in the RLQ7–T1006I–HLA-A2 complex, this bridging water molecule is expelled from the interface by small yet relevant movements in CDR1α that permit formation of two new compensating hydrogen bonds with the mutant peptide (Asp31α Oδ2–N P4 Ser and Asp31α Oδ2–N P5 Leu) and an additional hydrogen bond with HLA-A2 (Glu29α Oε2–Nζ2 Arg66 HLA-A2) (Fig. 4*C*).

The movements in CDR1α are likely the result of a 140° flip in the side chain of peptide residue P3 Gln caused by the T1006I mutation at P7. Although residues P3 and P7 are not in direct contact, the T1006I mutation induces movements in several HLA-A2 residues that are relayed to P3 Gln3 *via* HLA-A2 His74, which does contact P3 Gln3 (Fig. 4*A*). In total, the RLQ7–T1006I–HLA-A2 and RLQ7–RLQ–HLA-A2 complexes each contain nine direct hydrogen bonds linking TCR with peptide, as well as a comparable number of van der Waals contacts between TCR and peptide (62 and 68, respectively) (Tables S3 and S4), in agreement with the similar stabilities of the two complexes.

### Conformational changes in RLQ7 upon binding pMHC

**Figure 5:**
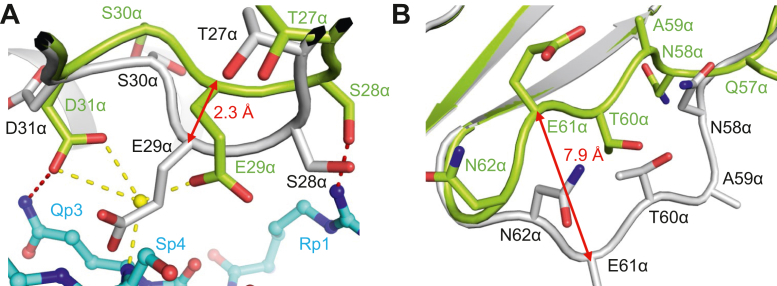
**Conformational changes in TCR RLQ7 upon binding RLQ-HLA-A2.***A*, superposition of TCR RLQ7 in unbound form and in complex with RLQ–HLA-A2 showing shifts in CDR1α residues 27 to 30 (unbound RLQ7, *gray*; bound RLQ7, *green*; RLQ, *cyan*). *Yellow sphere* is an interfacial water molecule. Hydrogen bonds are *red dashed lines*. Water-mediated hydrogen bonds are *yellow dashed lines*. Double-headed *red arrow* indicates region of structural rearrangements induced by binding to RLQ–HLA-A2. *B*, structural differences in Vα residues 57 to 62 of TCR RLQ7 associated with RLQ–HLA-A2 binding (unbound RLQ7, *gray*; bound RLQ7, *green*). CDR1α, complementarity-determining region 1α; TCR, T cell receptor.

To assess ligand-induced conformational changes in TCR RLQ7, we determined its structure in unbound form to 2.80 Å resolution (Table S1). Superposition of the VαVβ domains of free RLQ7 onto those in complex with RLQ–HLA-A2 revealed structural differences in CDR1α and in Vα residues 57 to 62. The CDR1α loop underwent small yet relevant movements (rmsd in α-carbon positions of 1.7 Å for residues 27–30) that enable CDR1α to engage the RLQ peptide *via* two direct and three water-mediated hydrogen bonds and 10 van der Waals contacts (Fig. 5*A*). CDR1α Glu29 showed the largest individual displacement (2.3 Å in its α-carbon position). Vα residues 57 to 62, which are distant from the TCR–pMHC interface, were in unambiguous electron density in both free and bound RLQ7. The 57–62 loop adopts markedly different main-chain conformations in the two structures, with rmsd in α-carbon positions of 4.7 Å (Fig. 5*B*). Vα Glu61 showed the largest individual displacement (7.9 Å in its α-carbon position). Whether this structural rearrangement is a consequence of RLQ–HLA-A2 binding or simply reflects a degree of malleability in Vα is unclear.

## Discussion

Immune escape by SARS-CoV-2 VOCs is a major concern in managing the COVID-19 pandemic (34). Extensive studies have been carried out to characterize the effects of mutations in the spike protein on recognition by neutralizing antibodies (35, 36). By contrast, the mechanisms employed by SARS-CoV-2 VOCs to evade T cell surveillance are less well documented (25). In general, viruses can escape T cell killing by interrupting one or more of several processes: antigen processing, peptide binding to MHC, or TCR recognition of pMHC. Emerging evidence indicates that SARS-CoV-2 utilizes each of these strategies.

The SARS-CoV-2 accessory proteins and ORF3a, ORF7a, and ORF8 downregulate MHC class I expression on infected cells to avoid elimination by cytotoxic T cells (37, 38). Several studies have identified mutations in T cell epitopes that impair epitope loading onto MHC class I molecules (22, 39, 40). In one report, mutations in HLA-A∗02:01– and HLA-B∗40:01–restricted spike epitopes resulted in weaker binding to the HLA, leading to reduced recognition by CD8^+^ T cells (22). Another report identified mutations in spike epitopes of VOCs that significantly diminished T cell responses in convalescent and vaccinated individuals by destabilizing epitope interactions with HLA-A∗02:01 and HLA-A∗24:02 (40). However, these studies did not provide evidence of dissemination of T cell escape variants in the wild, as might be expected if the variants conferred a selective advantage to the virus.

Mutations in T cell epitopes have also been identified that disrupt SARS-CoV-2 recognition by TCRs (24, 26). In particular, the P272L mutation in the HLA-A∗02:01–restricted YLQ spike epitope was found to have arisen in >100 different SARS-CoV-2 lineages, including VOCs, indicating transmission (24). Moreover, the P272L variant was not recognized by >175 individual YLQ-specific TCRs isolated from COVID-19 CPs and vaccinees, suggesting that this variant escapes from T cell responses.

At the same time, several mechanisms make T cell responses resilient to immune evasion. Because T cell responses to SARS-CoV-2 target multiple epitopes simultaneously (14, 41, 42, 43, 44, 45, 46, 47), it is unlikely that any single mutation can radically influence the overall magnitude of the response (48). In addition, the ability of most peptides to elicit many TCRs ensures broad and robust T cell responses that would not be possible if a single peptide could only elicit a few TCR clonotypes (49). Indeed, the number of T cell clones recognizing a particular SARS-CoV-2 epitope correlates with the persistence of the response to that epitope (9). Moreover, a mutated epitope that cannot bind to one HLA allele may often be presented by another HLA allele (50).

The restricted clonal, and therefore structural, diversity of YLQ-specific TCRs may facilitate viral escape and explain the transmission of the P272L mutation in multiple SARS-CoV-2 lineages (24, 26, 27, 28). By contrast, the clonal diversity of RLQ-specific TCRs should enable them to more readily circumvent epitope mutations. Whereas YLQ-specific TCRs engage YLQ–HLA-A2 *via* a common docking mode using nearly identical TRAV12-1 or TRAV12-2 gene segments and shared CDR3 motifs (24, 26, 27, 28), we have shown here that RLQ-specific TCRs can employ different α/β chain combinations to bind RLQ–HLA-A2 in structurally different ways. This structural diversity enables some RLQ-specific TCRs (e.g. RLQ7) to accommodate the T1006I mutation, even though others (e.g. RLQ3) cannot, thereby reducing the likelihood of extensive spread of this variant in the wild. The structures of the TCR RLQ7 and RLQ3 complexes with RLQ–HLA-A2 indicate that the successful epitope variant targeting of RLQ7 is likely achieved through its broader engagement of peptide and MHC residues, *versus* a more restricted focus on the peptide and its C-terminal portion (which contains the T1006 residue), for RLQ3.

A recently developed clinical-stage SARS-CoV-2 vaccine that utilizes a set of T cell epitopes (51) underscores the importance of epitope selection and T cell responses in vaccine design. Epitopes such as RLQ, which is highly conserved and associated with structurally diverse TCR targeting that can reduce the chance of immune evasion, may be useful components of prospective T cell epitope-based vaccines.

## Experimental procedures

### Protein preparation

The isolation of RLQ-specific TCR RLQ7 from COVID-19 CPs was described previously (16). Soluble TCR RLQ7 for structure determination was produced by *in vitro* folding from inclusion bodies expressed in *Escherichia coli*, as described previously for other SARS-CoV-2–specific TCRs (26). Codon-optimized genes encoding the TCR α (1–206) and β (1–245) chains were synthesized and cloned into the expression vector pET22b (GenScript). An interchain disulfide (CαCys160–CβCys172) was engineered to increase the folding yield of TCR RLQ7 αβ heterodimers. The mutated α and β chains were expressed separately as inclusion bodies in BL21(DE3) *E. coli* cells (Agilent Technologies). Bacteria were grown at 37 °C in LB medium to A_600_ = 0.6 to 0.8 and induced with 1 mM IPTG. After incubation for 3 h, the bacteria were harvested by centrifugation and resuspended in 50 mM Tris–HCl (pH 8.0) containing 0.1 M NaCl and 2 mM EDTA. Cells were disrupted by sonication. Inclusion bodies were washed with 50 mM Tris–HCl (pH 8.0) and 5% (v/v) Triton X-100, then dissolved in 8 M urea, 50 mM Tris–HCl (pH 8.0), 10 mM EDTA, and 10 mM DTT. For *in vitro* folding, the TCR α (45 mg) and β (35 mg) chains were mixed and diluted into 1 L folding buffer containing 5 M urea, 0.4 M L-arginine–HCl, 100 mM Tris–HCl (pH 8.0), 3.7 mM cystamine, and 6.6 mM cysteamine. After dialysis against 10 mM Tris–HCl (pH 8.0) for 72 h at 4 °C (buffer swapped at 48 h), the folding mixture was concentrated 20-fold and dialyzed against 50 mM MES buffer (pH 6.0) to precipitate misfolded protein. The supernatant was dialyzed overnight at 4 °C against 20 mM Tris–HCl (pH 8.0), 20 mM NaCl. Disulfide-linked TCR RLQ7 was purified using sequential Superdex 200 (20 mM Tris–HCl (pH 8.0), 20 mM NaCl) and Mono Q (20 mM Tris–HCl (pH 8.0), 0 to 1.0 M NaCl gradient) FPLC columns (GE Healthcare).

Soluble HLA-A2 loaded with RLQ peptide (RLQSLQTYV) or T1006I peptide (RLQSLQIYV) peptide was prepared by *in vitro* folding of *E. coli* inclusion bodies as described (52). Correctly folded RLQ–HLA-A2 and T1006I–HLA-A2 complexes were purified using consecutive Superdex 200 (20 mM Tris–HCl (pH 8.0), 20 mM NaCl) and Mono Q columns (20 mM Tris–HCl (pH 8.0), 0 to 1.0 M NaCl gradient).

### Crystallization and data collection

For crystallization of TCR–pMHC complexes, TCR RLQ7 was mixed with RLQ–HLA-A2 or RLQ-T1006I–HLA-A2 in a 1:1 ratio at a concentration of 7 mg/ml. Crystals were obtained at room temperature by vapor diffusion in hanging drops. The RLQ7–RLQ–HLA-A2 complex crystallized in 0.1 M Tris–HCl (pH 8.5), 0.01 M trimethylamine–HCl, and 15% (w/v) PEG 6000. Crystals of the RLQ7–T1006I–HLA-A2 complex grew in 0.1 M Tris–HCl (pH 8.5) and 15% (w/v) PEG 6000. Crystals of unbound RLQ7 were obtained in 0.02 M sodium phosphate monobasic, 0.1 M Bis-Tris propane (pH 7.5), and 20% PEG 3350. Before data collection, all crystals were cryoprotected with 20% (w/v) glycerol and flash-cooled. X-ray diffraction data were collected at beamline 23-ID-B of the Advanced Photon Source, Argonne National Laboratory. Diffraction data were indexed, integrated, and scaled using the program HKL2000 (53). Data collection statistics are shown in Table S1.

### Structure determination and refinement

Before structure determination and refinement, all data reductions were performed using the CCP4 software suite (54). Structures were determined by molecular replacement with the program Phaser (55) and refined with Phenix (56). The models were further refined by manual model building with Coot (57) based on 2*F*_o_ – *F*_c_ and *F*_o_ – *F*_c_ maps. The α chain of TCR 38-10 (PDB accession code 6VRN) (52), the β chain of TCR 1E6 (5C0B) (58), and p53R175H–HLA-A2 (6VR5) (52) with the CDRs and peptide removed were used as search models to determine the orientation and position of the RLQ7–RLQ–HLA-A2 complex. The orientation and position parameters of RLQ7–T1006I–HLA-A2 were obtained using the coordinates of the RLQ7–RLQ–HLA-A2 complex as a search model. The TCR component of the RLQ7–RLQ–HLA-A2 complex was used as a search model to determine the coordinates of unbound RLQ7. Refinement statistics are summarized in Table S1. Contact residues were identified with the CONTACT program (54) and were defined as residues containing an atom 4.0 Å or less from a residue of the binding partner. The PyMOL program (https://pymol.org/) was used to prepare figures.

### Computational sequence and structural analysis

Computational mutagenesis and ΔΔG calculations were performed using the interface mutagenesis protocol in Rosetta v.2.3 (59), as performed previously (26), with command line flags included to perform side chain minimization before and after modeling of mutant residue (“-min_interface -int_chi”). Hotspots are defined as alanine substitutions with predicted binding ΔΔ*G* ≥ 1.0 in Rosetta Energy Units. Interface buried interface surface area calculations were performed using the NACCESS program (60) by subtracting the calculated surface area of the complex from the separately calculated surface areas of the TCR and pMHC components from the complex structure. Interface buried surface area values from the TCR3d database (33) are based on the same calculation method.

## Data availability

Atomic coordinates and structure factors have been deposited in the Protein Data Bank under accession codes 8GOP (RLQ7), 8GOM (RLQ7–RLQ–HLA-A2), and 8GON (RLQ7–T1006I–HLA-A2).

## Supporting information

This article contains supporting information (26, 54, 59).

## Conflict of interests

The authors declare that they have no conflicts of interest with the content of this article.
