# Supplementary material for: Structural insights into protection against a SARS-CoV-2 spike variant by T cell receptor diversity
Source: J Biol Chem. 2023 Feb 17;299(4):103035. doi: 10.1016/j.jbc.2023.103035 (PMC9934920; doi:10.1016/j.jbc.2023.103035)
Supplement: Supplemental data [file mmc1.docx]

**Supporting Information**

**Structural insights into protection against a SARS-CoV-2 spike variant by T cell receptor (TCR) diversity**

Daichao Wu, Grigory A. Efimov, Apollinariya V. Bogolyubova, Brian G. Pierce, and Roy A. Mariuzza

**Supporting Figures 1 and 2**

**Supporting Tables 1–5**

**
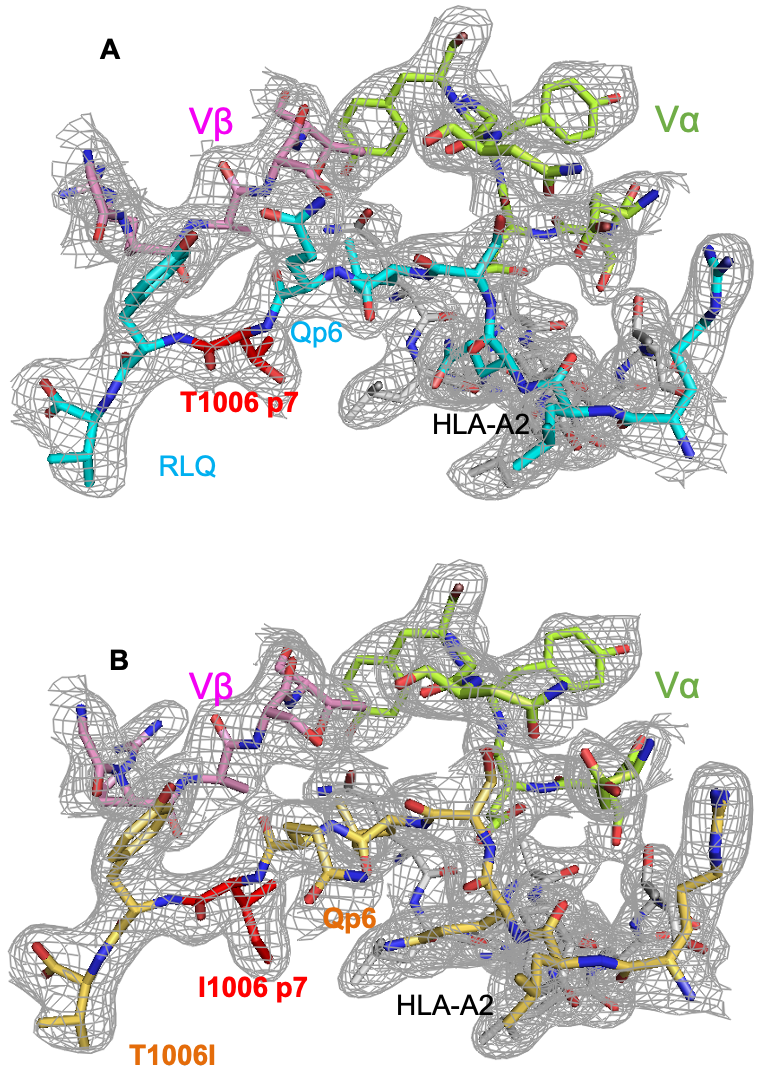
**

**Figure S1. Electron density in the interfaces of the RLQ7–RLQ–HLA-A2 and RLQ7–T1006I–HLA-A2 complexes.** *A*, electron density in the interface of the RLQ7–RLQ–HLA-A2 complex. Density from the final 2*F*_o_ – *F*_c_ map at 2.78 Å resolution is contoured at 1σ. *B*, electron density in the interface of the RLQ7–T1006I–HLA-A2 complex. Density from the final 2*F*_o_ – *F*_c_ map at 2.60 Å resolution is contoured at 1σ. Carbon atoms are green (RLQ7 α chain), pink (RLQ7 β chain), cyan (RLQ peptide), or yellow (T1006I peptide). Residues P7 Thr in wild-type RLQ and P7 Ile in the T1006I variant are shown in red.

**
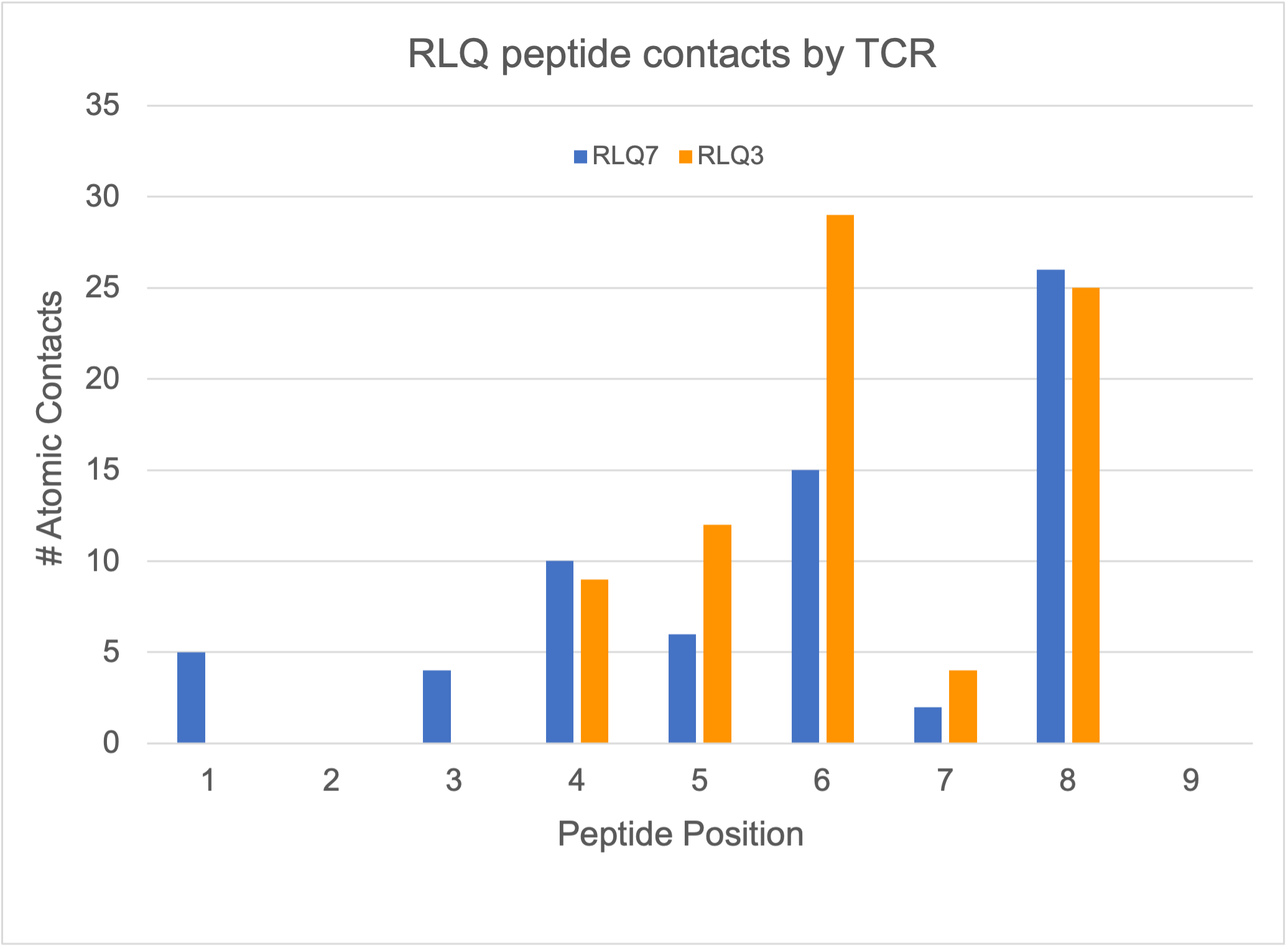
Figure S2. TCR RLQ7 and RLQ3 contacts with RLQ peptide by peptide position.** Atom contact counts are based on a 4.0 Å distance cutoff, and reflect the values shown in **Table S4**.

**Table S1. Data collection and refinement statistics**

|  | RLQ7–RLQ–HLA-A2 | RLQ7–T1006I– HLA-A2 | RLQ7 |
| --- | --- | --- | --- |
| PDB accession code | 8GOM | 8GON | 8GOP |
| **Data collection** |  |  |  |
| Resolution range (Å) | 46.6–2.78 (2.88–2.78) | 48.2–2.6 (2.7–2.6) | 34.8–2.8 (2.9–2.8) |
| Space group | *P* 65 2 2 | *P* 65 2 2 | P 1 21 1 |
| Unit cell parameters | 147.4, 147.4, 178.5  90, 90, 120 | 147.4, 147.4, 178.8  90, 90, 120 | 52.2, 68.1, 63.9,  90, 90.2, 90 |
| Total reflections^a^ | 524,012 (32,521) | 897,986 (74,112) | 38,168 (3,761) |
| Unique reflections^a^ | 29,103 (2,669) | 35,792 (3,505) | 10,990 (1,107) |
| Multiplicity^a^ | 18.0 (12.2) | 25.1 (21.1) | 3.5 (3.4) |
| Completeness (%)^a^ | 99.2 (93.6) | 99.9 (100.0) | 94.5 (95.8) |
| Mean *I*/σ(*I*)^a^ | 19.0 (2.4) | 22.0 (2.6) | 9.4 (1.7) |
| Wilson *B* factor (Å^2^) | 51.0 | 55.6 | 38.5 |
| *R*_merge_^a,b^ | 0.125 (0.638) | 0.113 (0.692) | 0.353 (0.560) |
| CC1/2 | 0.994 (0.911) | 0.994 (0.942) | 0.842 (0.647) |
|  |  |  |  |
| **Refinement** |  |  |  |
| Reflections used in refinement^a^ | 29,093 (2,668) | 35,759 (3,504) | 10,539 (1,078) |
| *R*_work_^c^ | 0.196 (0.287) | 0.200 (0.260) | 0.238 (0.353) |
| *R*_free_^c^ | 0.246 (0.344) | 0.249 (0.297) | 0.297 (0.472) |
| No. of protein atoms | 6,566 | 6,561 | 3,491 |
| No. of waters | 133 | 117 | 117 |
| Protein residues | 819 | 819 | 443 |
| r.m.s.d. from ideality |  |  |  |
| Bond lengths (Å) | 0.007 | 0.011 | 0.005 |
| Bond angles (^o^) | 1.16 | 1.40 | 1.02 |
| Ramachandran plot statistics |  |  |  |
| Favored (%) | 97.0 | 95.8 | 96.6 |
| Allowed (%) | 3.0 | 4.2 | 3.4 |
| Disallowed (%) | 0 | 0 | 0 |
| Rotamer outliers (%) | 1.4 | 2.1 | 1.6 |
| Clashscore | 7.9 | 7.6 | 9.7 |
| Average *B* factor (Å^2^) | 47.3 | 56.1 | 35.5 |
| Protein | 47.4 | 57.7 | 35.7 |
| Waters | 42.4 | 51.7 | 29.5 |

^a^Values in parentheses correspond to the highest resolution shell.

^b^*R*_merge_ = ∑|*I*_j_ – <*I*>|/∑*I*_j_, where *I*_j_ is the intensity of an individual reflection and <*I*> is the average intensity of that reflection.

^c^*R*_work_ (*R*_free_) = ∑||*F*_o_| – |*F*_c_||/∑|*F*_o_|; 5.0% of data were used for *R*_free_.

**Table S2. Interactions between** **TCRs and HLA-A2**

| HLA-A2 | RLQ7–RLQ–HLA-A2 | | RLQ7–T1006I–HLA-A2 | | RLQ3–RLQ–HLA-A2 | |
| --- | --- | --- | --- | --- | --- | --- |
|  | Hydrogen bonds | Van der Waals contacts | Hydrogen bonds | Van der Waals contacts | Hydrogen bonds | Van der Waals contacts |
| α1 |  |  |  |  |  |  |
| G62H |  | N97α(2) |  |  |  |  |
| R65H | T98α(Oγ1) R65H(Nη2) | D56β(1),  S58β(1) | T98α(Oγ1) R65H(Nη2) | D56β(1),  S58β(1) |  |  |
| K66H |  | E29α(1),  N97α(2) | E29α(Oε2) R66H(Nζ2) | E29α(3),  N97α(2) |  | N92α(2) |
| A69H |  | I55β(1) |  |  |  |  |
| Q72H |  | V53β(1),  P54β(2),  I55β(1) |  | P54β(1),  I55β(2) |  |  |
| R75H |  | V53β(1) |  | V53β(3) |  |  |
| V76H |  | N50β(2),  N51β(2),  A53β(3 |  | N50β(2),  N51β(2),  V53β(2) |  | N49β(3) |
| T80H |  | N51β(1) |  | N51β(1) | N49β(Nδ2) T80H(Oγ1) | N49β(2) |
| α2 |  |  |  |  |  |  |
| H145H |  |  |  |  |  | L94β(2) |
| K146H |  | R98β(3) |  | R98β(2) |  | G26β(1),  L94β(3),  G96β(2) |
| W147H |  | R98β(1) |  |  | G96β(O) W147H(Nε1) | G96β(3) |
| A149H |  | R98β(2) |  | R98β(1) |  | L94β(1),  D101β(2) |
| A150H |  | R98β(1),  D102β(1) |  | R98β(4),  D102β(1) | R48α(Nη2) A150H(O) | R48α(3),  A100β(1),  D101β(5) |
| H151H |  | Y54α(1) |  | Y54α(2) | S51α(Oγ) H151H(Nε2) | I50α(1), S51α(4) |
| E154H |  | Y54α(4) |  | Y54α(5) |  | I50α(1) |
| Q155H | Y33α(Oη) Q155H(Nε2),  T101β(Oγ1)Q155H(Nε2),  T101β(Oγ1) Q155H(Oε1) | D31α(1),  Y33α(6),  Y54α(1),  T101β(3) | Y33α(Oη) Q155H(Nε2),  T101β(Oγ1) Q155H(Nε2),  T101β(Oγ1) Q155H(Oε1) | D31α(1),  Y33α(9),  Y54α(1),  T101β(3) | E31α(Oε2) Q155H(Nε2),  R48α(Nη1) Q155H(Nε2) | E31α(3),  R48α(3),  I50α(3),  F91α(4) |
| A158H | S30α(Oγ) A158H(O) | S30α(3),  Y54α(1) | S30α(Oγ) A158H(O) | S30α(4),  Y54α(1) |  |  |
| Y159H |  | S30α(1),  D31α(2) |  | S30α(1) |  |  |
| G162H |  | S30α(2) |  | S30α(2) |  |  |
| T163H | S30α(N) T163H(Oγ1),  S30α(Oγ) T163H(Oγ1),  S30α(Oγ) T163H(N), | E29α(2),  S30α(4) | S30α(N) T163H(Oγ1),  S30α(Oγ) T163H(Oγ1),  S30α(Oγ) T163H(N) | E29α(3),  S30α(5) |  |  |

Contact residues were identified with the CONTACT program (54). Hydrogen bonds were calculated using a cut-off distance of 3.5 Å. The cut-off distance for van der Waals contacts was 4 Å.

**Table S3. Interactions between** **TCRs and RLQ peptide**

| RLQ | RLQ7–RLQ–HLA-A2 | | RLQ7–T1006I–HLA-A2 | | RLQ3–RLQ–HLA-A2 | |
| --- | --- | --- | --- | --- | --- | --- |
|  | Hydrogen bonds | Van der Waals contacts | Hydrogen bonds | Van der Waals contacts | Hydrogen bonds | Van der Waals contacts |
| p1R | S28α(Oγ) p1R(Nη1) | S28α(3),  E29α(1) | S28α(O) p1R(Nη1) | S28α(1),  E29α(1) |  |  |
| p3Q | D31α(Oδ2) p3Q(Nε2) | D31α(3) |  | D31α(3) |  |  |
| p4S | G96α(O) p4S(Oγ)  N97α(N) p4S(Oγ)  N97α(Oδ1) p4S(Oγ) | E29α(2),  G96α(2),  N97α(3) | D31α(Oδ2) p4S(N),  G96α(O) p4S(Oγ),  N97α(N) p4S(Oγ),  N97α(Oδ1) p4S(Oγ) | D31α(6),  E29α(2),  G96α(3),  N97α(4) | N92α(Nδ2) p4S(Oγ) | S29α(4), F91α(2)  N92α(2) |
| p5L |  | D31α(1),  S100β(3),  T101β(2) | D31α(Oδ2) p5L(N) | D31α(3),  A99β(1),  S100β(1),  T101β(1) |  | F91α(9),  N96α(2)  A100β(1 |
| p6Q | S100β(Oγ) p6Q(N), S100β(N) p6Q(O),  Y48β(Oη) Q6p(Nε2) | S100β(4),  A99β(3),  Y48β(4),  I55β(1) | S100β(Oγ) p6Q(N), S100β(N) p6Q(O) | S100β(2),  A99β(3) | F91α(O) p6Q(Nε2),  N93α(O) p6Q(Nε2),  G95α(N) p6Q(Oε1),  N96α(Nδ2) p6Q(N),  N96α(Oδ1) p6Q(N),  N96α(Nδ2) p6Q(O),  N96α(Oδ1)p6Q(Nε2) | F91α(2),  N93α(3),  A94α(2),  G95α(3),  N96α(9)  A97β(3) |
| p7T/I |  | R98β(2) |  | R98β(3) |  | G96β(4) |
| p8Y | R98β(O) p8Y(N) | R98β(2),  G97β(3),  N50β(16),  I55β(4) | R98β(O) p8Y(N) | R98β(1),  G97β(3),  N50β(14),  I55β(2) | G96β(O) p8Y(N),  G96β(O) p8Y(O) | Q48β(11),  V53β(1),  G95β(3),  G96β(6),  G98β(2) |

Contact residues were identified with the CONTACT program (54). Hydrogen bonds were calculated using a cut-off distance of 3.5 Å. The cut-off distance for van der Waals contacts was 4 Å.

**Table S4. Number of contacts between** **TCR and peptide or MHC**

| **Peptide residue or chain total** | **RLQ7** | **RLQ7 T1006I** | **RLQ3** |
| --- | --- | --- | --- |
| p1 | 5 | 3 | 0 |
| p2 | 0 | 0 | 0 |
| p3 | 4 | 3 | 0 |
| p4 | 10 | 19 | 9 |
| p5 | 6 | 7 | 12 |
| p6 | 15 | 6 | 29 |
| p7 | 2 | 3 | 4 |
| p8 | 26 | 21 | 25 |
| p9 | 0 | 0 | 0 |
| Total peptide | 68 | 62 | 79 |
| Total MHC | 68 | 74 | 55 |

Counts shown are atomic contacts with TCR at 4.0 Å distance cutoff for individual peptide residues (p1–p9), or full peptide or MHC. “RLQ7” and “RLQ3” correspond to the RLQ7–RLQ–HLA-A2 and RLQ3–RLQ–HLA-A2 interfaces, respectively, and “RLQ7 T1006I” corresponds to RLQ7 in complex with the T1006I RLQ mutant (RLQ7–T1006I–HLA-A2).

**Table S5. Computational alanine scanning of the RLQ peptide.**

| **Mutation** | **RLQ3 ΔΔ*G*** | **RLQ7 ΔΔ*G*** |
| --- | --- | --- |
| R1A | 0 | 0.3 |
| L2A | 0 | 0 |
| Q3A | 0 | **1.6** |
| S4A | -0.1 | 0.6 |
| L5A | **1** | 0.7 |
| Q6A | **2.3** | **2.1** |
| T7A | 0.2 | 0.2 |
| Y8A | **1.9** | **2.4** |
| V9A | 0 | 0 |

Computational alanine scanning mutagenesis of the RLQ epitope was performed in Rosetta (59) using the RLQ3–RLQ–HLA-A2 and RLQ7–RLQ–HLA-A2 complex structures to predict binding affinity changes (ΔΔ*G*s) for TCRs RLQ3 and RLQ7. ΔΔ*G* values are in values of Rosetta Energy Units, comparable to energies in kcal/mol, and values in bold reflect predicted hotspot residues (ΔΔ*G* ≥ 1.0). RLQ3 ΔΔ*G* values are from our previously reported analysis of the RLQ3–RLQ–HLA-A2 interface (26).
